# Exploring potential roles for the interaction of MOM1 with SUMO and the SUMO E3 ligase-like protein PIAL2 in transcriptional silencing

**DOI:** 10.1371/journal.pone.0202137

**Published:** 2018-08-09

**Authors:** Qiu-Yuan Zhao, Xin-Jian He

**Affiliations:** National Institute of Biological Sciences, Beijing, China; RIKEN Center for Sustainable Resource Science, JAPAN

## Abstract

The CHD3-like chromatin remodeling protein MOM1 and the PIAS-type SUMO E3 ligase-like protein PIAL2 are known to interact with each other and mediate transcriptional silencing in Arabidopsis. However, it is poorly understood whether and how the interaction is involved in transcriptional silencing. Here, we demonstrate that, while the PIAL2 interaction domain (PIAL2-IND) is required for PIAL2 dimerization, MOM-PIAL2 interaction, and transcriptional silencing, a transgene fusing the wild-type MOM1 protein with the PIAL2 protein defective in PIAL2-IND can completely restore transcriptional silencing in the *mom1/pial2* double mutant, demonstrating that the artificial fusion of MOM1 and PIAL2 mimics the *in vivo* interaction of these two proteins so that PIAL2-IND is no longer required for transcriptional silencing in the fusion protein. Further, our yeast two-hybrid assay identifies a previously unrecognized SUMO interaction motif (SIM) in the conserved MOM1 motif CMM3 and demonstrates that the SIM is responsible for the interaction of MOM1 with SUMO. Given that eukaryotic PIAS-type SUMO E3 ligases have a conserved role in chromatin regulation, the findings reported in this study may represent a conserved chromatin regulatory mechanism in higher eukaryotes.

## Introduction

In eukaryotic genomes, transposons, DNA repeats, and exogenous transgenes are usually subjected to transcriptional silencing through DNA methylation [1–3]. In Arabidopsis, DNA methylation is established by RNA-directed DNA methylation pathway at CG, CHG, CHH sites (H represents A, T and C) [1,3]. CG, CHG, and CHH methylation are predominantly maintained by the DNA methyltransferases MET1 [4], CMT3 [5], and CMT2 [6,7], respectively. However, transcriptional silencing regulators, such as the CHD3-like protein MOM1 [8], the Microrchidia (MORC) family proteins MORC1 and MORC6 [9–11], and proteins that are related to DNA replication and nucleosome assembly [12,13], are involved in transcriptional silencing indepently of alteration in DNA methylation. While DNA methylation-dependent transcriptional silencing has been extensively studied, how these DNA methylation-independent regulators mediate tanscriptional silencing needs to be investigated.

The *mom1* mutant was first identified by screening for mutants that release silencing of the hygromycin-resistant transgene [14]. At the whole genome level, MOM1 regulates transcriptional silencing without alteration of DNA methylation [15,16]. Mutation in NRPE1, the largest subunit of Pol V in the RdDM pathway, was identified as an enhancer of the *mom1* mutant by a forward genetic screen [17]. Our recent report showed that MOM1 cooperates with the RdDM pathway to regulate the silencing of their common target loci independently of alteration in DNA methylation [18]. The MOM1 protein contains an incomplete SNF2 domain, a nuclear localization domain, and three conserved MOM1 motifs: CMM1, CMM2, CMM3 [8]. While a complete SNF2 domain contains seven conserved motifs parted by a cleft, MOM1 only contains the second part [8]. The CMM2 motif itself is effective for silencing a subset of MOM1 target genomic loci that are co-regulated by the RdDM pathway [19]. However, little is known how the CMM2 domain is involved in transcriptional silencing.

Small ubiquitin-like modifier (SUMO) is known to regulate various biological processes, including development, defense, and stress response [20,21]. SUMO1 and SUMO2 are closely related to each other and belong to a subclass of the SUMO protein family in Arabidopsis [21]. The double mutants of SUMO1 and SUMO2 are embryonically lethal [21]. Our recent study demonstrated that SUMO1 and SUMO2 redundantly function in transcriptional silencing of transposons and repetitive DNA elements [22]. However, how SUMO is involved in transcriptional silencing remains elusive. SUMO can covalently modify other proteins through a cascade of reaction: activation of SUMO by E1 (SUMO-activating enzyme), conjugation of SUMO by E2 (SUMO-conjugating enzyme), and ligation of SUMO by E3 (SUMO ligase) [23]. In Arabidopsis, many nuclear proteins are covalently modified by SUMO as determined by proteomic analyses [24]. Besides the covalent modification, SUMO can non-covalently interact with other proteins through SUMO interaction motif (SIM) [25].

Our previous study demonstrated that two conserved PIAS-type SUMO E3 ligase-like proteins, PIAL1 and PIAL2 (PIAL1/2), interact with MOM1 and thereby mediate transcriptional silencing [18]. Although PIAL1 and PIAL2 have the SUMO E3 ligase activity [26], the activity is dispensable for their function in transcriptional silencing [18]. It is unknown whether and how the interaction of MOM1 and PIAL1/2 is involved in transcriptional silencing. MOM1 was shown to non-covalently interact with SUMO1 as identified by a yeast two-hybrid screen [25]. It remains to be determined whether MOM1 interacts with SUMO1 *in vivo*; if the interaction occurs, it is necessary to clarify whether and how the interaction contributes to transcriptional silencing.

Here, we generated a transgene fusing MOM1 with the PIAL2 protein harboring mutations in the conserved PIAL2 interaction domain (PIAL2-IND) and performed complementation testing, demonstrating that the fusion transgene can completely restore transcriptional silencing in the *mom1pial2* double mutant. The study suggests that PIAL2-IND is exclusively responsible for the interaction of PIAL2 with MOM1 and demonstrates that the fusion of PIAL2 with MOM1 mimics the interaction so that PIAL2-IND is dispensable for transcriptional silencing in the fusion protein. Further, our yeast two-hybrid assay identified a previously uncharacterized SIM in the conserved MOM1 domain CMM3 and demonstrated that the SIM is responsible for interaction with SUMO. The study provides an insight into the molecular mechanism for understanding how the CHD3-like chromatin remodeling protein MOM1 cooperates with the SUMO E3 ligase-like proteins to regulate transcriptional silencing. The mechanism revealed in this study may represent a conserved chromatin regulatory mechanism in higher eukaryotic organisms.

## Results

### Mutations in the PIAL2-IND domain impair PIAL2-MOM1 interaction and PIAL2 dimerization

**Fig 1:**
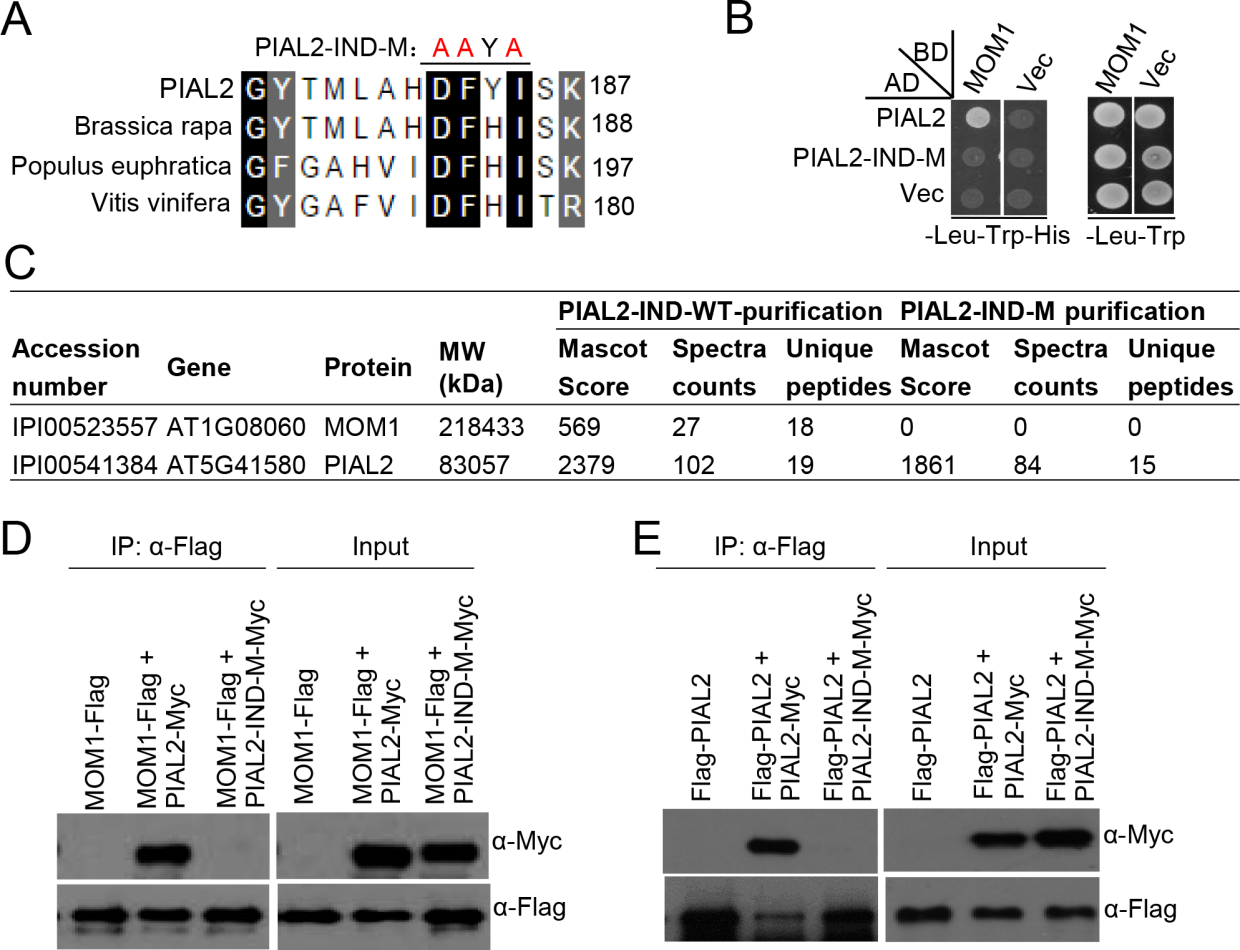
The IND domain of PIAL2 is responsible for MOM1-PIAL2 interaction and PIAL2 dimerization *in vivo*. **(A)** Schematic diagram of mutations in the IND domain of PIAL2. Black squares represent the conserved amino acids. PIAL2-IND-M represents the mutations in the IND domain (D182A/F183A/I185A, shown in red). **(B)** The interaction between MOM1 and PIAL2 or PIAL2-IND-M as determined by yeast two-hybrid assays. MOM1 was fused to GAL4-BD in the *pGBKT7* vector; PIAL2 and PIAL2-IND-M were fused to GAL4-AD in the *pGADT7* vector. “Vec” represents the empty *GAL-BD* or *GAL4-AD* vectors. **(C)** The IND mutations impair the interaction between PIAL2 and MOM1 as determined by affinity purification followed by mass spectrometric analysis. Protein extraction from transgenic plants separately harboring the wild-type *PIAL2* and *PIAL2-IND-M* transgene were subjected to affinity purification. **(D)** The interaction of MOM1-Flag with the wild-type PIAL2-Myc and the PIAL2-IND-M-Myc as determined by co-IP. **(E)** The interaction of Flag-PIAL2 with the wild-type PIAL2-Myc and the PIAL2-IND-M-Myc as determined by co-IP.

Our previous study indicated that the PIAS-type SUMO E3 ligase-like protein PIAL2 contains an interaction domain (IND) that can dimerize and interact with the conserved CMM2 domain of MOM1 as determined by an *in vitro* pull down assay [18]. However, the *in vivo* function of the IND domain needs to be examined. We compared the IND domains (143–201 amino acids) from the Arabidopsis PIAL2 and its plant orthologues in *Brassica rapa*, *Populus euphratica*, and *Vitis vinifera*, indicating that the IND domain is highly conserved in plants (S1 Fig). To determine the function of the IND domain *in vivo*, we mutated the highly conserved residues in the IND domain (IND-M, D182A/F183A/I185A; Fig 1A) and introduced the mutations into the full-length PIAL2 cDNA sequence for yeast two-hybrid assays. The result indicated that, while the wild-type PIAL2 interacts with MOM1, the mutations impair the interaction of PIAL2 and MOM1 (Fig 1B).

To determine whether the PIAL2-IND mutations affect the interaction of PIAL2 and MOM1 in Arabidopsis, we introduced the mutations into a construct carrying the full-length *PIAL2* genomic sequence fused with a 3’-terminal *Myc* tag and transformed the construct into Arabidopsis to obtain mutated *PIAL2* (*PIAL2-IND-M-Myc*) transgenic plants. We extracted proteins from the mutated and wild-type *PIAL2* transgenic plants and performed affinity purification with the anti-Myc antibody followed by mass spectrometric analysis. The result showed that MOM1 was co-purified with the wild-type PIAL2 but not with PIAL2-IND-M (Fig 1C), indicating that the IND mutation impairs the interaction of PIAL2 with MOM1 in Arabidopsis. To further confirm the effect of the IND mutations on the interaction of PIAL2 with MOM1, we crossed the wild-type and mutated *PIAL2-Myc* transgenic plants with *MOM1-Flag* transgenic plants, and extracted proteins from the F1 generation plants for co-immunoprecipitation (co-IP) assays. Our result indicated that the wild-type PIAL2-Myc protein but not the PIAL2-IND-M-Myc protein was precipitated with MOM1-Flag (Fig 1D), confirming that the PIAL2-IND domain is responsible for the interaction of PIAL2 and MOM1 *in vivo*.

Considering our previous study reporting that the PIAL2-IND domain can form a homo-dimer as determined by an *in vitro* assay [18], we wonder whether the IND mutations that disrupt the PIAL2-MOM1 interaction also affect the dimerization of PIAL2. We crossed the wild-type *PIAL2-Myc* and *PIAL2-IND-M-Myc* transgenic plants with transgenic plants carrying *PIAL2* fused with a 5’-terminal *Flag* tag (*Flag-PIAL2*). Using the F1 generation plants, we performed co-IP and demonstrated that the wild-type PIAL2-Myc but not the PIAL2-IND-M-Myc was co-immunoprecipitated with the Flag-PIAL2 protein (Fig 1E), demonstrating that the PIAL2-IND domain is required for both the PIAL2-MOM1 interaction and the PIAL2 dimerization *in vivo*.

### Artificial fusion of MOM1 with the PIAL2 protein defective in the IND domain is effective for transcriptional silencing

**Fig 2:**
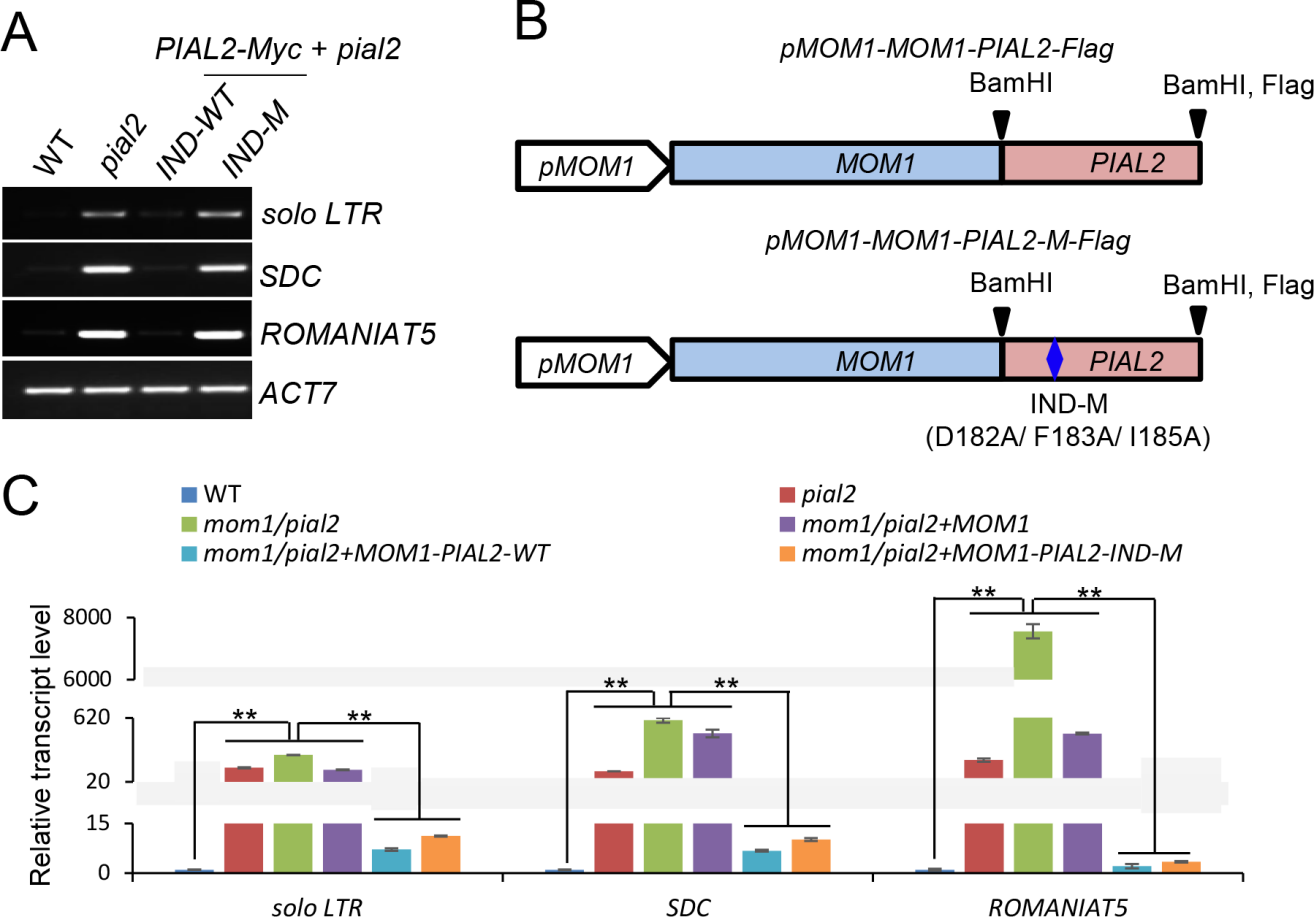
The IND domain of PIAL2 is involved in transcriptional silencing through interaction with MOM1. **(A)** Complementation of the silencing defect in the *pial2* mutant by wild-type and mutated *PIAL2* transgenes. The mutated *PIAL2* transgene contains *D182A/F183A/I185A* mutations in the *IND* domain of *PIAL2*. The expression of the PIAL2 target loci was examined by RT-PCR. *ACT7* was used as a control. **(B)** Schematic diagram of wild-type and mutated *MOM1-PIAL2* fusion genes. The wild-type and mutated *PIAL2* was fused to the 3’-terminal of the wild-type *MOM1* driven by the *MOM1* promoter through the BamHI restriction site. Both the fusion genes harbor a *Flag* tag in their 3’-terminals. **(C)** Complementation of the silencing defect in the *mom1/pial2* double mutant by *MOM1*, *MOM1-PIAL2*, and *MOM1-PIAL2-IND-M* transgenes. The expression of *solo LTR*, *SDC*, and *ROMANIAT5* was detected by qPCR. *ACT7* served as an internal control. Error bars are standard deviation (SD). *P < 0.05 or **P < 0.01 was determined by Student’s t test.

Our previous study showed that PIAL2 and MOM1 interact with each other and regulate transcriptional silencing at many common target genomic loci [18]. However, it is unknown whether the PIAL2-MOM1 interaction is required for the function of PIAL2 and MOM1 in transcriptional silencing. Considering the effect of the PIAL2-IND mutations on the interaction of PIAL2 with MOM1, we predicted that the IND mutations should affect transcriptional silencing if the PIAL2-MOM1 interaction is required for transcriptional silencing. To detect the effect of the IND mutations on transcriptional silencing, we transformed the wild-type *PIAL2* construct and the mutated *PIAL2* construct harboring the IND mutations (D182A/ F183A/ I185A) into the *pial2* mutant plants for complementation testing. The expression levels of the wild-type and mutated *PIAL2* transgenes were comparable as determined by western blotting (S2 Fig). The genomic loci *solo LTR*, *SDC*, and *ROMANIAT5* were previously demonstrated to be co-regulated by PIAL2 and MOM1 in Arabidopsis [18]. Our RT-PCR results showed that these loci were silenced in the wild-type plants and indicated that the silencing was released in the *pial2* mutant (Fig 2A). While the wild-type *PIAL2* transgene fully restored the silencing of these loci, the mutated *PIAL2* transgene failed to restore the silencing (Fig 2A), demonstrating that the interaction of PIAL2 and MOM1 is required for the function of PIAL2 in transcriptional silencing.

Although the IND domain is required for the PIAL2-MOM1 interaction, we cannot exclude the possibility that the IND domain may have some other molecular roles that are responsible for the function of PIAL2 in transcriptional silencing. If the IND domain is exclusively involved in the PIAL2-MOM1 interaction, artificial fusion of PIAL2 and MOM1 may be functional even when the IND domain is mutated. To determine whether the IND domain is involved in transcriptional silencing through interaction with MOM1, we generated constructs in which the wild-type *MOM1* was fused with the wild-*type PIAL2* (*MOM1-PIAL2*) and with the mutated *PIAL2* harboring the *IND* mutations (*MOM1-PIAL2-IND-M*) under the control of the *MOM1* promoter (Fig 2B). The constructs were independently transformed into the *mom1/pial2* double mutant for complementation testing. We selected transgenic lines that showed comparable expression levels between wild-type and mutated *MOM1-PIAL2* fusion genes (S3 Fig), and examined whether the *IND* mutations affect transcriptional silencing. Our quantitative PCR experiment indicated that the *MOM1-PIAL2* fusion transgene restored the silencing of *solo LTR*, *SDC*, and *ROMANIAT5* to the wild-type level in the *mom1/pial2* double mutant, whereas the *MOM1* transgene without the fusing *PIAL2* was unable to restore the silencing (Fig 2C). This result demonstrates that the *MOM1-PIAL2* fusion gene can combine the function of *MOM1* and *PIAL2* in transcriptional silencing. Compared to the wild-type *MOM1-PIAL2* fusion gene, the *MOM1-PIAL2* fusion gene harboring the *IND* mutations was able to restore the silencing as well (Fig 2C), indicating that the *IND* mutations do not affect the function of the *MOM1-PIAL2* fusion gene in transcriptional silencing. We infer that the IND domain of PIAL2 is involved in transcriptional silencing exclusively through interaction with MOM1.

### Mutations in the CMM2 domain impair MOM1 dimerization and partially affect the interaction of MOM1 with PIAL1 and PIAL2

**Fig 3:**
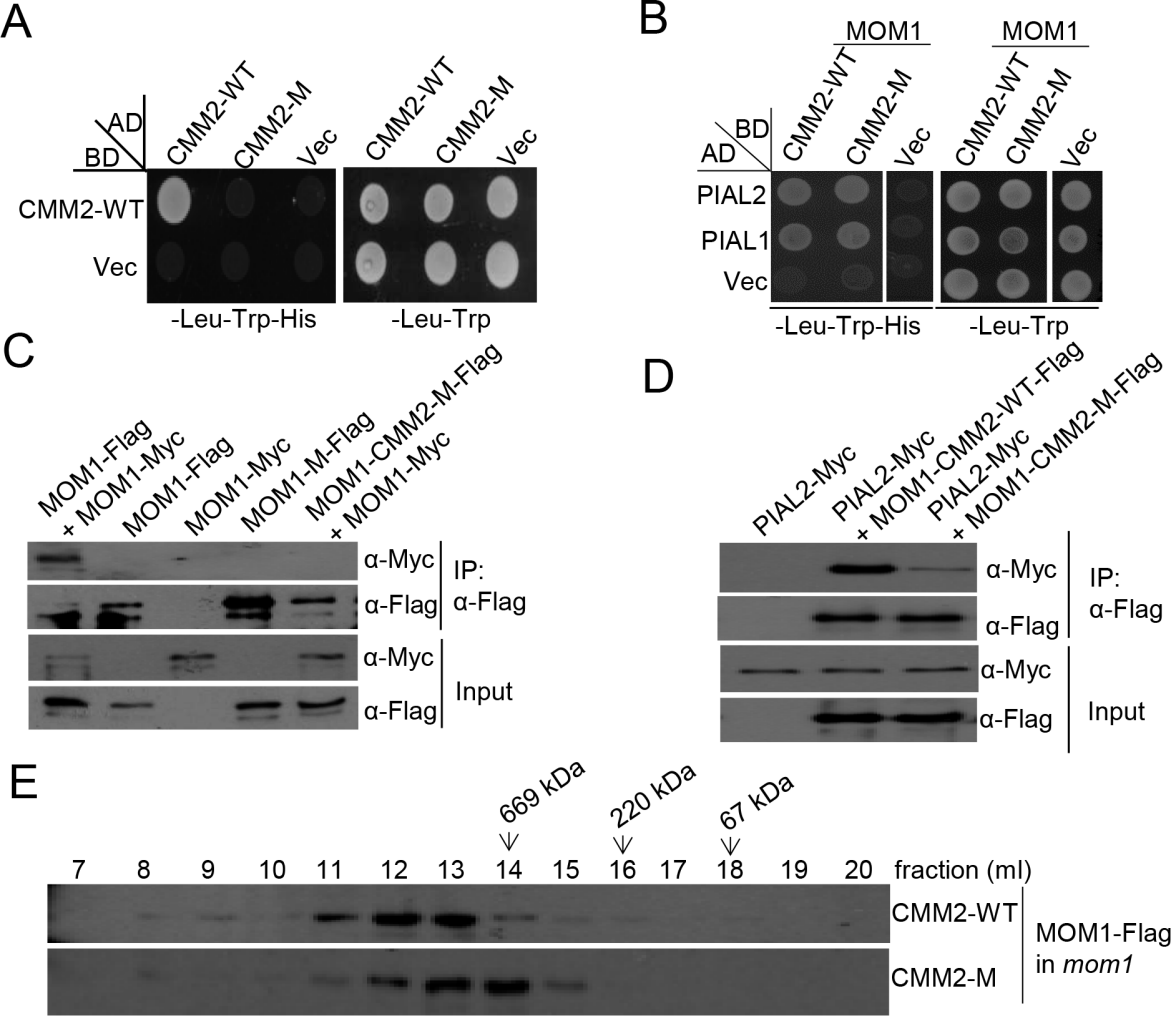
Mutations in the CMM2 domain affect MOM1 dimerization and MOM1-PIAL2 interaction. **(A)** The L1761D/L1765D mutations in the CMM2 domain disrupted the CMM2 dimerization as determined by yeast two-hybrid assays. The wild-type CMM2 was fused to GAL4-BD; the wild-type and the mutated CMM2 were fused to GAL4-AD. “Vec” represents the empty *GAL-BD* or *GAL4-AD* vectors. **(B)** The full-length MOM1 harboring the CMM2 mutations still interacts with PIAL1 and PIAL2 in yeast. The wild-type and mutated MOM1 were fused to GAL4-BD; PIAL1 and PIAL2 were fused to GAL4-AD. “Vec” represents the empty *GAL-BD* or *GAL4-AD* vectors. **(C)** The mutations in the CMM2 domain disrupt MOM1 dimerization *in vivo* as determined by co-IP. The wild-type and mutated *MOM1-Flag* were introduced into transgenic plants harboring the *MOM1-Myc* transgene by crossing. F1 generation plants were subjected to co-IP. **(D)** The mutations in the CMM2 domain partially impair the interaction of MOM1 with PIAL2 *in vivo* as determined by co-IP. The wild-type and mutated *MOM1-Flag* were separately introduced into transgenic plants harboring *PIAL2-Myc* transgene by crossing. **(E)** The CMM2 mutations in the CMM2 domain of MOM1 are required for MOM1 complex formation *in vivo* as determined by gel filtration assays. Protein exaction from transgenic plants harboring the wild-type *MOM1-Flag* and the mutated *MOM1-CMM2-M-Flag* in the *mom1* background was eluted on Superose 6 (10/300 GL) column. The fractions were subjected to western blotting. The arrows mean the fractions corresponding to the standard proteins of 67, 220, 669 kDa.

The CMM2 domain of MOM1 was previously reported to form a homo-dimer and play an essential role in transcriptional silencing [19]. Interestingly, our previous study indicated that the CMM2 domain also interacts with PIAL1/2 [18]. As previously reported [19], the CMM2 mutations L1761D/L1765D were known to impair the CMM2 dimerization. However, it is unknown whether the CMM2 mutations affect the interaction of the CMM2 domains with PIAL1/2. We introduced the mutations into the CMM2 domain and performed yeast two-hybrid assays to test the effect of the mutations on the CMM2 dimerization and the interaction of the CMM2 domain with PIAL1/2. The yeast two-hybrid result showed that the CMM2 mutations impaired the CMM2 dimerization but not the interaction of the CMM2 domain with PIAL1/2 (Fig 3A and 3B).

We previously demonstrated that MOM1 is dimerized *in vivo* [18]. To investigate whether the CMM2 mutations impair the MOM1 dimerization *in vivo*, we performed co-IP assays. The CMM2 mutations were introduced into the full-length *MOM1* sequence fused with a 3’-terminal *Flag* tag. By crossing, the mutated *MOM1-CMM2-M-Flag* and wild-type *MOM1-Flag* transgenes were introduced into the Arabidopsis plants harboring the *MOM1-Myc* transgene. Using the F1 generation plants, we performed co-IP assays with anti-Flag antibody. The result showed that the CMM2 mutation impaired the MOM1 dimerization in Arabidopsis (Fig 3C). To test whether the CMM2 mutation affects the interaction of MOM1 with PIAL2, we introduced the mutated *MOM1-CMM2-M-Flag* and wild-type *MOM1-Flag* transgenes into the *PIAL2-Myc* transgenic plants by crossing. Our co-IP experiment indicated that PIAL2-Myc was co-immunoprecipitated by the wild-type MOM1-Flag and to a lesser extent by the mutated MOM1-CMM2-M-Flag (Fig 3D). Thus, although the CMM2 mutations do not affect the interaction of the CMM2 domain with PIAL2 as determined by yeast two-hybrid assays (Fig 3B), the CMM2 mutations impair the interaction of MOM1 with PIAL2 in Arabidopsis.

MOM1 and PIAL1/2 were known to form a high molecular weight complex *in vivo* [18]. To determine whether the CMM2 domain of MOM1 is required for forming the complex, we transformed the wild-type *MOM1-Flag* and the mutated *MOM1 -Flag* harboring the CMM2 mutations into the *mom1* mutant and extracted proteins from the transgenic seedlings to perform gel filtration assay (Fig 3E). Our previous study indicated that the wild-type MOM1-Myc protein forms a high molecular weight complex *in vivo* and demonstrated the formation of the complex is disrupted in the *pial1/2* mutant [18]. As expected, the wild-type MOM1-Flag protein forms a high molecular weight complex *in vivo* as determined by the gel filtration assay in this study (Fig 3E). Although the mutated MOM1-Flag protein also forms a complex, the size of the complex is significantly smaller than that of the wild-type MOM1-Flag complex (Fig 3E). This finding supports the notion that the CMM2 mutations affect the MOM1 dimerization and thereby impair the formation of the high molecular weight complex. The presence of the smaller complex from the mutated *MOM1-Flag* transgenic plants is consistent with the co-IP results indicating that the CMM2 mutations partially affect the MOM1-PIAL2 interaction (Fig 3D). We predict that the weak MOM1-PIAL1/2 interaction in the mutated MOM1-Flag transgenic plants may be responsible for forming the smaller complex.

### The CMM2 mutations affect the function of MOM1 in transcriptional silencing

Considering that the CMM2 mutations affect the formation of the high molecular weight MOM1-PIAL1/2 complex, we predicted that the CMM2 mutations might also affect the function of MOM1 in transcriptional silencing. Thus, we introduced the CMM2 mutations into the full-length *MOM1* transgene for complementation testing in Arabidopsis. As previously reported [19], the MOM1 target genomic loci were divided into two classes based on whether the silencing of the loci is dependent on RNA-directed DNA methylation (RdDM). Class I loci, such as *ROMANIAT5* and *TSI*, are up-regulated in the *mom1* mutant but not or weakly up-regulated in RdDM mutants; Class II loci, such as *solo LTR* and *SDC*, are co-up-regulated in both the *mom1* and RdDM mutants.

**Fig 4:**
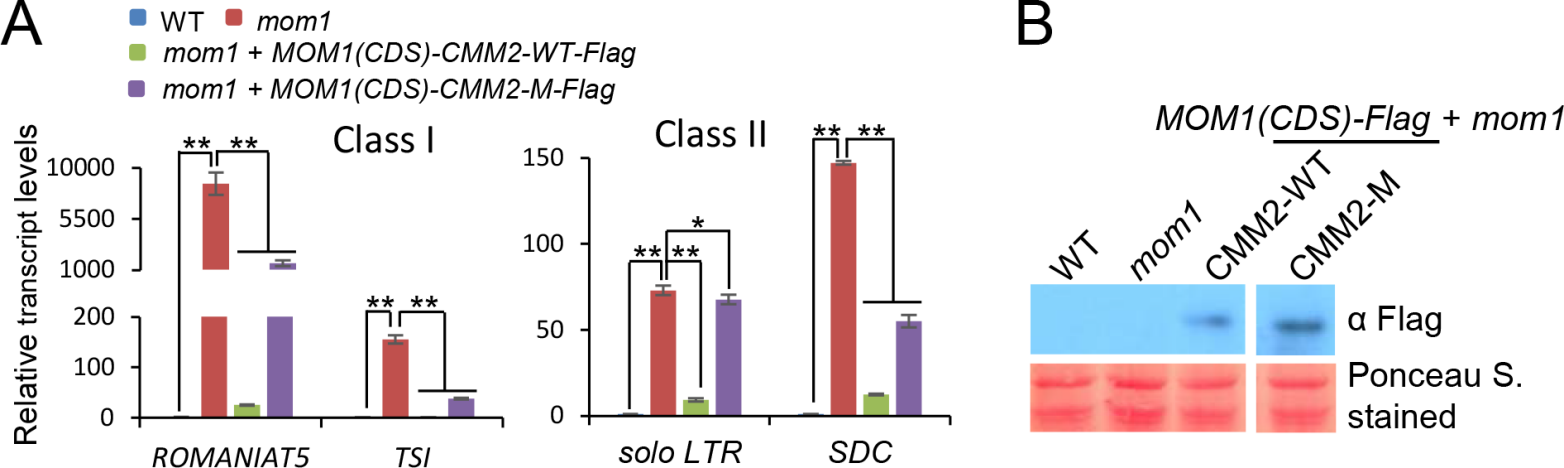
Mutations in the CMM2 domain of MOM1 affect transcriptional silencing. **(A)** Silencing of indicated loci was largely restored by the wild-type *MOM1-Flag* transgene but was only slightly restored by the *MOM1-Flag* transgene harboring the mutations in the CMM2 domain. Class I loci are only regulated by MOM1, including *ROMANIAT5* and *TSI*; Class II loci are co-regulated by MOM1 and RdDM pathway, including *solo LTR* and *SDC*. *ACT7* was used as an internal control. Error bars are SD. *P < 0.05 or **P < 0.01 was determined by Student’s t test. **(B)** Expression levels of the wild-type and mutated *MOM1* transgenes in *mom1* mutant as determined by western blotting. The transgenic lines were used to determine the effect of the CMM2 mutations on transcriptional silencing. Rubisco stained by Ponceau S was shown as a loading control.

Our quantitative PCR results indicated that the wild-type *MOM1* transgene markedly restored the silencing of both Class I and II types of MOM1 target loci in the *mom1* mutant (Fig 4A). The CMM2 mutations in the full-length *MOM1* transgene significantly affect transcriptional silencing (Fig 4A). The expression level of the mutated *MOM1* transgene was comparable to that of the wild-type *MOM1* transgene (Fig 4B). Therefore, failure of the mutated *MOM1* transgene to restore transcriptional silencing in the *mom1* mutant is caused by the CMM2 mutations in the mutated *MOM1* transgene. Given the effect of the CMM2 mutations on the formation of the high molecular weight complex and on the function of MOM1 in transcriptional silencing, we conclude that the formation of the high molecular weight complex is required for the function of MOM1 in transcriptional silencing. Of note, our quantitative PCR results indicated that the *MOM1* transgene harboring the CMM2 mutations weakly restore transcriptional silencing in the *mom1* mutant. Thus, although the CMM2 mutations completely disrupted the MOM1 dimerization, the disruption of the MOM1 dimerization does not completely suppress the MOM1 function in transcriptional silencing.

### MOM1 and PIAL2 interact with SUMO as determined by yeast two-hybrid assays

The PIAS-type SUMO E3 ligase-like protein PIAL2 was shown to have a conserved SUMO interaction motif (SIM) that is necessary for its SUMO E3 ligase activity [26]. However, it is unknown whether the SIM is responsible for the interaction of PIAL2 with SUMO. We demonstrated that PIAL2 was able to interact with SUMO2 as determined by yeast two-hybrid assays (S4A Fig). The SIM domain of PIAL2 was responsible for the interaction of PIAL2 with SUMO2 but not with MOM1 (S4B and S4C Fig). These results demonstrated that the SIM domain is specifically responsible for the interaction of PIAL2 with SUMO as determined by yeast two-hybrid assays. However, our previous study indicated that the SIM mutations did not affect the function of PIAL2 in transcriptional silencing, implying that PIAL2 is involved in transcriptional silencing independently of the interaction of PIAL2 and SUMO.

**Fig 5:**
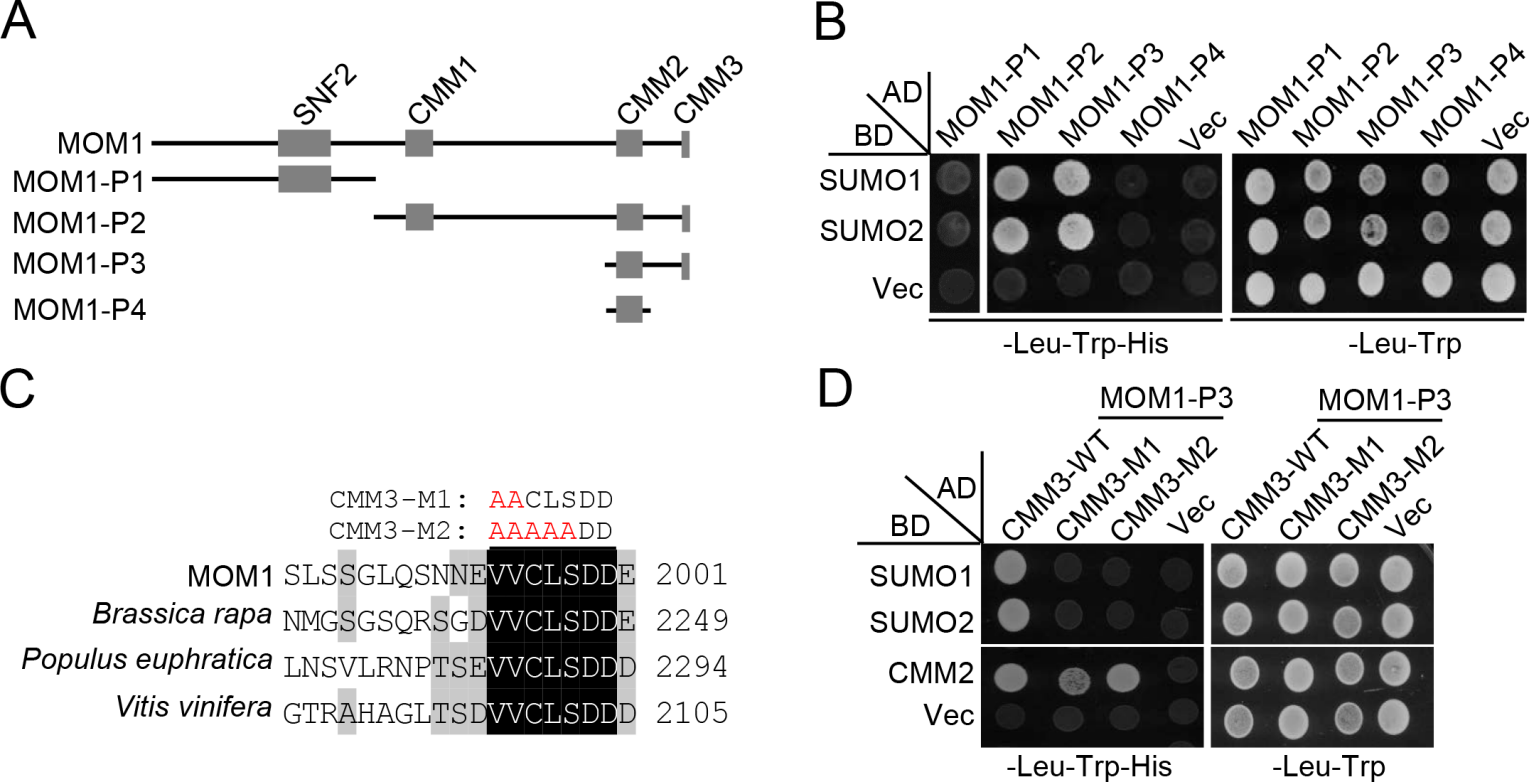
The CMM3 domain of MOM1 is responsible for SUMO1 and SUMO2 interaction as determined by yeast two-hybrid assays. **(A)** Schematic diagram of full-length and various truncated versions of MOM1 used in yeast two-hybrid assays. P1, 1–832 aa; P2, 798–2001 aa; P3, 1660–2001 aa; P4, 1660–1860 aa. **(B)** Interaction of full-length and truncated versions of MOM1 with SUMO1, SUMO2 as determined by yeast two-hybrid assays. Full-length and truncated versions of MOM1 were fused to GAL4-AD, and SUMO1 and SUMO2 was fused to GAL4-BD. “Vec” represents the empty *GAL-BD* or *GAL4-AD* vectors. **(C)** Alignment of the CMM3 domains of Arabidopsis MOM1 and its orthologues from other plants, including *Brassica rapa*, *Populus euphratica* and *Vitis vinifera*. The amino acids highlighted by black and gray backgrounds indicated that the amino acids are completely and partially conserved, respectively. CMM3-M1 (V1994A/V1995A, shown in red) and CMM3-M2 (V1994A/V1995A/ C1996A/L1997A/S1998A, shown in red) represent two versions of mutations in the CMM3 domain of MOM1. **(D)** Mutations in the CMM3 domain disrupt the interaction between MOM1-P3 and SUMO1 or SUMO2 as determined by yeast two-hybrid assays. The wild-type and mutated MOM1-P3 were fused with GAL4-AD. SUMO1, SUMO2, and CMM2 were fused with GAL4-BD. “Vec” represents the empty *GAL-BD* or *GAL4-AD* vectors.

MOM1 was previously shown to interact with SUMO1 as determined by a yeast two-hybrid screen [25]. To determine which domain in MOM1 is required for the interaction of MOM1 with SUMO, we generated a series of truncated MOM1 fragments (MOM1-P1 ~ MOM1-P4) for yeast two-hybrid assays (Fig 5A). Our result demonstrated that MOM1-P2 and MOM1-P3 but not MOM1-P1 and MOM1-P4 interact with SUMO1 and SUMO2 (Fig 5B), suggesting that the conserved C-terminal CMM3 is necessary for the interaction of MOM1 with SUMO proteins. Although CMM3 was previously identified as a conserved domain in MOM1 [19], its function was not yet understood. By alignment of MOM1 and its orthologues in plants, we observed that the CMM3 is highly conserved in plants and contains a putative SUMO interaction motif (SIM: VVCLS) (Fig 5C; S1 Fig). To determine whether the SIM is indeed responsible for the interaction of MOM1 with SUMO1 and SUMO2, we carried out yeast two-hybrid assays by using two different mutated MOM1-P3 fragments harboring CMM3-M1 (V1994A/V1995A) and CMM3-M2 (V1994A/V1995A/C1996A/L1997A/ S1998A) mutations (Fig 5C). The result showed that both the two versions of the CMM3 mutations impaired the interaction of MOM1-P3 with SUMO1 and SUMO2 (Fig 5D), demonstrating that the SIM in the CMM3 domain is responsible for the interaction of MOM1 with SUMO1 and SUMO2. However, the CMM3-M1 and CMM3-M2 mutations in the MOM1-P3 fragments did not affect the interaction with the CMM2 domain (Fig 5D). These results suggest that the SIM in the CMM3 domain is responsible for the interaction of MOM1 with SUMO1 and SUMO2 but not for the MOM1 dimerization.

### The interaction of MOM1 with SUMO is not required for transcriptional silencing

**Fig 6:**
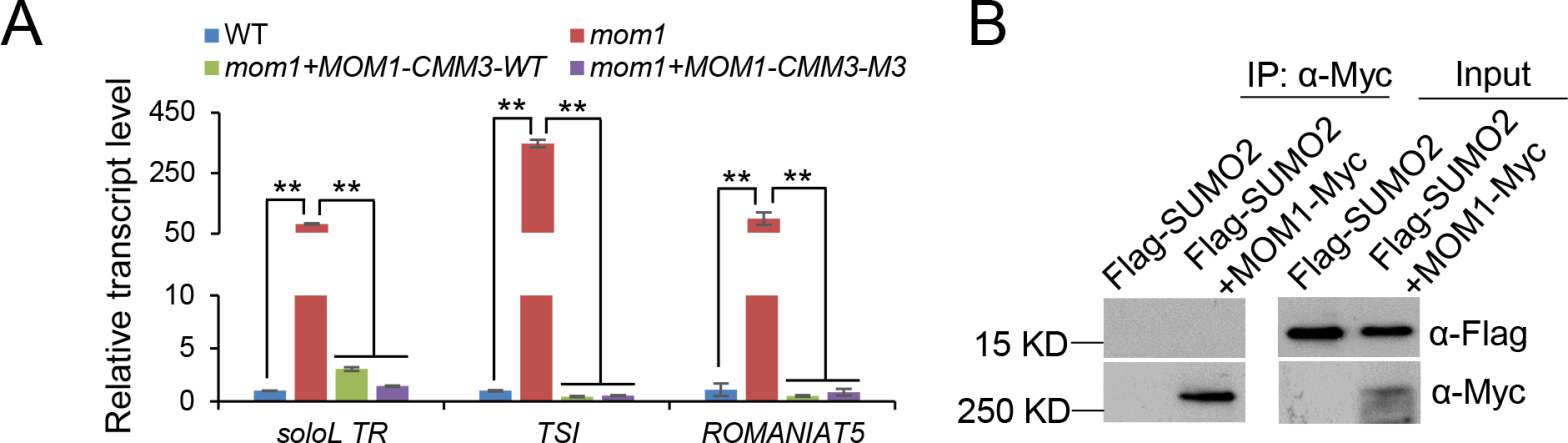
The interaction of MOM1 with SUMO proteins is dispensable for transcriptional silencing. **(A)** The SIM in the CMM3 domain of MOM1 is not required for transcriptional silencing. As determined by qPCR, the silencing of *solo LTR*, *TSI*, and *ROMANIAT5* was restored by the *MOM1* transgene harboring the CMM3 mutations as well as by the wild-type transgene. *ACT7* served as an internal control. Error bars are SD. *P < 0.05 or **P < 0.01 was determined by Student’s t test. **(B)** SUMO2 was not co-purified with MOM1 as determined by co-IP. *MOM1-Myc* transgene was introduced into transgenic plants harboring *Flag-SUMO2* transgene by crossing. F1 generation plants were subjected to co-IP.

To determine whether the CMM3 domain is necessary for transcriptional silencing, we introduced the CMM3 mutations (V1994A/V1995A/L1997A/S1998A) into the full-length *MOM1* construct and tested whether the mutations affect the function of MOM1 in transcriptional silencing in Arabidopsis. We carried out western blotting to compare the wild-type and mutated *MOM1-Flag* expression level in their transgenic lines and selected the transgenic lines that showed comparable protein levels for complementation testing (S5 Fig). Our complementation test indicated that the mutated *MOM1* transgene complemented the silencing of the MOM1 target loci as well as the wild-type *MOM1* transgene (Fig 6A). The results suggest that even though MOM1 interacts with SUMO1 and SUMO2 as determined by yeast two-hybrid assays, the SIM of MOM1 is dispensable for the function of MOM1 in transcriptional silencing. To determine whether MOM1 interacts with SUMO in Arabidopsis, we crossed the *MOM1-Myc* transgenic plants with transgenic plants harboring a native promoter-driven *SUMO2* fused with a 5’-terminal *Flag* tag (*Flag-SUMO2*). The seedlings from the F1 generation were used for co-IP. The result indicated that MOM1 was not able to co-purify with SUMO2 (Fig 6B), suggesting that the MOM1 cannot interact with SUMO2 in Arabidopsis. Thus, although the CMM3 domain of MOM1 is shown to interact with SUMO1 and SUMO2 as determined by yeast two-hybrid assays, it is necessary to investigate the function of the conserved CMM3 domain in future.

## Discussion

Although the CHD3-like chromatin remodeling protein MOM1 is known to act as a transcriptional silencing regulator, it is poorly understood how MOM1 is involved in transcriptional silencing. In the *mom1* mutant, DNA methylation, histone modifications, and heterochromatin condensation are not significantly affected while transcriptional silencing is disrupted, suggesting that MOM1 regulates transcriptional silencing through an uncharacterized mechanism [14–16]. Mutations in RdDM components were previously identified by screening for enhancers of the *mom1* mutant [17], suggesting that MOM1 can function together with RdDM components in transcriptional silencing. It was thought that MOM1 could act at a downstream step of RdDM to mediate the repressive H3K9 dimethylation at some specific RdDM target loci [27]. However, at the whole genome level, many genomic loci that were regulated by MOM1 but not by RdDM were identified [17,18]. How MOM1 is involved in transcriptional silencing at these genomic loci needs to be studied. From a yeast two-hybrid screen, MOM1 was shown to interact with SUMO [25]. However, it is unknown whether the interaction of MOM1 and SUMO is involved in transcriptional silencing. Our previous study demonstrated that MOM1 interacts with the PIAS-type SUMO E3 ligase-like proteins PIAL1/2 and form a large molecular weight complex [18]. Although we have demonstrated that PIAL1/2 are involved in transcriptional silencing [18], it is necessary to investigate whether the interaction of PIAL1/2 with MOM1 is required for transcriptional silencing.

Our previous study demonstrated that the IND domain of PIAL2 could interact with PIAL2 and MOM1 *in vitro* [18]. Here, we introduced mutations in the IND domain of PIAL2 and demonstrated that the mutations disrupted the interaction of PIAL2 with MOM1 and the PIAL2 dimerization as determined by both the yeast two-hybrid assay and the co-IP experiment. Further, we introduced the mutations into the *PIAL2* transgene and demonstrated that the mutations impaired the function of PIAL2 in transcriptional silencing. These results strongly suggest that the integrity of MOM1-PIAL2 complex is necessary for its function in silencing. We predict that the MOM1-PIAL2 complex may serve as an adaptor complex to recruit some downstream silencing regulators. We artificially fused the wild-type *MOM1* with the mutated *PIAL2* harboring the mutations in the IND domain and found that the fusion gene completely complemented defects in transcriptional silencing in the *pial2mom1* double mutant, whereas the *MOM1* transgene without the fusion with the mutated *PIAL2* can only partially complement the defects in transcriptional silencing. The result suggests that in addition to the IND domain, some other domains of the PIAL2 protein are necessary for the function of PIAL2 in transcriptional silencing. In addition to the IND domain, PIAL2 contains putative SIM and RING domains, which are required for the SUMO E3 ligase activity. Our previous studies have demonstrated that, although the putative SIM domain of PIAL2 is required for the interaction of PIAL2 with SUMO as determined by yeast two-hybrid assays, it is dispensable for the function of PIAL2 in transcriptional silencing [18]. The putative RING domain of PIAL2 is highly similar with the zf-MIZ domain, which was primarily identified in the transcription factor MIZ-1 in animals and was shown to be responsible for binding specific DNA sites [28]. We speculate that the RING domain of PAL2 may be responsible for recruitment of the MOM1-PIAL1/2 complex to chromatin.

In a previous study [19], a *miniMOM1* transgene that encodes the CMM2 domain and the nuclear localization signal was reported to be effective for silencing RdDM-dependent MOM1 target loci, whereas it was less effective for silencing RdDM-independent MOM1 target loci. The CMM2 mutations in the *miniMOM1* transgene were previously reported to significantly affect the silencing of RdDM-dependent MOM1 target loci but only have a weak effect on RdDM-independent MOM1 target loci [19]. Our result indicates that the CMM2 mutations in the full-length *MOM1* transgene significantly affect the silencing of both RdDM-dependent and–independent MOM1 target loci. As shown by the previous study [19], the *miniMOM1* transgene only has a limited role in silencing RdDM-independent MOM1 target loci. Thus, the weak effect of the CMM2 mutations in the *miniMOM1* transgene is most likely due to the limited role of the *miniMOM1* transgene in the silencing of RdDM-independent MOM1 target loci.

We previously demonstrated that MOM1 not only forms a dimer but also interacts with PIAL2 *in vivo* [18]. The CMM2 domain of MOM1 is responsible for the dimerization and the interaction with PIAL2 as determined by *in vitro* pull down assays [18]. Our results show that mutations in the CMM2 domain impair the MOM1 dimerization and disrupt the function of MOM1 in transcriptional silencing (Figs 3A, 3C and 4A). These results are consistent with the previous finding that the CMM2 domain is involved in transcriptional silencing [8]. Thus, our study implies that the CMM2 domain mediates MOM1 dimerization and thus facilitates transcriptional silencing. However, we demonstrate that the CMM2 mutation does not disrupt the MOM1 function in silencing completely (Fig 4A). Our co-IP experiment indicated that, although the CMM2 mutations completely disrupted the MOM1 dimerization, it only partially impaired the interaction of MOM1 with PIAL2 (Fig 3D). We predict that the remaining MOM1-PIAL2 interaction may be responsible for the residual function of the mutated MOM1 in transcriptional silencing.

There are four Arabidopsis SUMO E3 ligases: SIZ1, HPY2, PIAL1, and PIAL2, which contain an SP-RING motif required for binding specific substrates and SUMO conjugating enzymes directly [20,29]. These ligases are involved in various biological processes, including development and stress responses [30–34]. By identifying sumoylated proteins *in vivo*, some proteins related to chromatin, transcription, and RNA processes were identified as sumoylated substrates [35,36]. Our recent study confirmed that the Su(var)39-related protein SUVR2 is sumoylated *in vivo* and the sumoylation is required for the interaction of SUVR2 with the chromatin-remodeling proteins CHR19 and CHR27 [22]. In addition to the proteins that are covalently modified by SUMO, some proteins are shown to interact with SUMO non-covalently. In animals, SUMO was reported to mediate transcriptional regulation by non-covalently interacting with its substrates [37,38]. Here, we identify a SIM motif in the conserved CMM3 domain of MOM1 and demonstrate that the SIM motif directly interacts with SUMO as determined by yeast two-hybrid assay. However, the SIM in the CMM3 is dispensable for the function of MOM1 in transcriptional silencing. This result is consistent with our previous study reporting that the SUMO ligase activity of the MOM1-interacting protein PIAL2 is not required for transcriptional silencing [18]. Although the interaction of MOM1 with SUMO was detected as examined by yeast two-hybrid assays, the interaction was not detected by our co-IP experiment (Fig 6B). Some transcription factors in mammals have a low sumoylation level, but the low level of sumoylation is required for transcriptional regulation [39]. Thus, we cannot entirely exclude the possibility that MOM1 may covalently or non-covalently interact with SUMO at low levels or at specific growth conditions, which has not been detected by our co-IP experiment. In Arabidopsis. sumoylation was shown to be activated by heat and oxidative stresses [40]. Transposon silencing can be released under stress conditions such as heat shock [41,42]. Therefore, although the interaction of SUMO with MOM1 and PIAL2 is dispensable for transcriptional silencing as examined in our experimental conditions, it may play regulatory roles in transcriptional silencing and other biological processes under some specific development stages and environment conditions. Further studies are required to clarify whether and how SUMO is involved in the function of MOM1 and PIAL2 in transcriptional silencing.

## Materials and methods

### Plant materials and growth conditions

The Arabidopsis seedlings were grown on MS (Murashige and Skoog) medium [43] under long-day conditions (16 h light and 8 h dark) for 12 days and were then transplanted into soils. The Arabidopsis plants used in this study were in the Col-0 ecotype. The T-DNA insertion mutants *mom1* (SALK_141293) and *pial2* (SALK_043892) were obtained from Arabidopsis Biological Resource Center (ABRC). We crossed *mom1* to *pial2* and identified the *mom1/pial2* double mutant in the F2 generation plants. The genomic sequences of *MOM1* and *PIAL2* driven by corresponding native promoters were cloned into *pCAMBIA1305* or *pRI909* with 3’-terminal *Flag* or *Myc* tags. The constructs were transformed into plants through the agrobacterium directed flower-dipping method [44]. Point mutations were introduced into the corresponding constructs by site-directed mutagenesis. For the *PIAL2* and *SUMO2* constructs harboring a 5’-terminal *Flag* tag, the corresponding promoter was cloned in front of the *Flag* tag. For generating a *MOM1-PIAL2* fusion gene, *MOM1* was fused with 3’-terminal *PIAL2* followed by a *Flag* tag. All constructs were verified by sequencing. Primers used for generating the constructs were listed in S1 Table.

### Analyses of RNA levels

Arabidopsis seedlings grown on MS medium for 12 days and 50 mg seedlings were subjected to RNA extraction with 1 ml of Trizol (Sigma) reagent. 500 μl of chloroform was added to remove proteins. The mixture was centrifuged at 12,000 rpm for 15 min at 4°C. The supernatant was added with 500 μl of isopropanol to precipitate RNA. The RNA was washed with 1 ml of 75% of ethanol. According to the manufacturer's instruction, 20 μg RNA in total 20 μl volume was subjected to reverse transcription by 5×All-In-One RT MasterMix (ABM). We diluted 20 μl of cDNA to 80 μl and used 4 μl of cDNA for qPCR by SYBR Green Master Mix (Kapa Biosystems) in each reaction. *ATC7* was served as an internal control.

### Affinity purification, mass spectrometric analysis, co-IP, and gel filtration

Given the observation that the protein levels of MOM1 and PIAL2 are higher in flowers than in seedlings, we isolated proteins from flowers for affinity purification. Five grams of flowers were harvested and subjected to protein extraction in 12 ml of lysis buffer (50 mM Tris-HCl, pH = 7.6, 150 mM NaCl, 5 mM MgCl_2_, 10% glycerol, 0.1% Nonidet P-40 [Amresco], 1 mM DL-dithiothreitol [Inalco], 1 mM phenylmethanesulfonyl fluoride (PMSF), 1 proteinase inhibitor cocktail tablet/50 ml [Roche]). The protein extraction was incubated with 80 μl anti-Flag affinity gel (Sigma) for 2.5 h at 4°C, washed 4 times with lysis buffer, and eluted with 3×Flag peptide (Sigma). The input proteins and immunoprecipitated proteins were boiled, subjected to SDS-PAGE and the gel was stained with ProteinSilver Silver Stain Kit (Sigma). The protein bands were cut and used for mass spectrometric analysis. For co-IP, after affinity purification, the input proteins and immunoprecipitated proteins were boiled and subjected to SDS-PAGE and western blotting. For gel filtration, 0.5 g seedlings were grounded to powder and subjected to protein extraction. The sample was subjected to centrifugation, and the supernatant was filtrated by 0.22 μm filter. Each of 500 μl samples was loaded onto Superose 6 (10/300 GL) column (GE Healthcare). The eluate proteins were collected per 500 μl and subjected to SDS-PAGE and western blotting.

### Yeast two-hybrid assay

The CDS sequences of the full-length and truncated forms of *MOM1*, *PIAL1*, and *PIAL2* were separately cloned into the *pGADT7* and *pGBKT7* vectors, fused with 5’-terminal *GAL4-AD* and *GAL4-BD* through the One-Step Cloning Kit (Vazyme Biotech). The yeast strains AH109 and Y187 were separately transformed with 200 ng of the *pGADT7* and *pGBKT7* constructs. The strains transformed by the *pGADT7* and *pGBKT7* constructs were incubated on synthetic dropout medium without Leu and Trp, respectively. The positive clones from the different synthetic dropout mediums were subjected to mating with each other in the YPD medium overnight. Seven microliter of each mating strain was spotted on synthetic dropout medium without Leu and Trp, and then the positive clones were transferred to grow on new synthetic dropout medium without Leu and Trp (-Leu-Trp), and synthetic dropout medium without Leu, Trp and His (-Leu-Trp-His) with the addition of 3 mM 3-amino-1, 2, 4-triazole to reduce background growth. Growth on synthetic dropout medium without Leu, Trp and His indicates that the two fusion proteins interact with each other.

## Accession numbers

Sequence data can be obtained in the Arabidopsis Genome Initiative database by the accession numbers below: MOM1 (AT1G08060), PIAL1 (AT1G08910), PIAL2 (AT5G41580), SUMO1 (AT4G26840), SUMO2 (AT5G55160).

## Supporting information

S1 Fig Analysis of the Arabidopsis PIAL2 protein. **(A)** Schematic representation of the Arabidopsis PIAL2 protein. **(B)** Alignment of PIAL2 and its homologs in *Brassica rapa*, *Populus trichocarpa and Vitis vinifera*. The IND domain of PIAL2 is from 143 to 201 amino acids. The SIM domain is from 425 to 428 amino acids.
(PDF)

S2 Fig Determination of the expression of the wild-type and mutated *PIAL2* transgenes. Both the wild-type (*PIAL2-WT*) and mutated (*PIAL2-IND-M*) transgenes were driven by the native *PIAL2* promoter and tagged by Myc epitope in their C-terminals. The expression of the transgenes was detected by western blotting. Rubisco stained by Ponceau S was shown as a loading control.
(PDF)

S3 Fig Determination of the expression of the *MOM1-Flag* transgene, the wild-type *MOM1-PIAL2* fusion transgene, and the *MOM1-PIAL2* fusion transgene harboring the mutations in the *IND* domain of *PIAL2*. The transgenes were introduced into the *mom1/pial2* double mutant and their expression was determined by western blotting. Rubisco stained by Ponceau S was shown as a loading control.
(PDF)

S4 Fig The SIM domain of PIAL2 is responsible for PIAL2 interaction with SUMO2 but not with MOM1. **(A)** PIAL2 interacts with SUMO2 as determined by yeast two-hybrid assays. PIAL1 and PIAL2 were fused with GAL4-AD. SUMO2 were fused with GAL4-BD. “Vec” represents the empty *GAL-BD* or *GAL4-AD* vectors. **(B)** Schematic representation of mutations in the SIM domain of PIAL2. The mutated Val, Phe, Asp, and Leu residues of PIAL2 are in blue. The Ala residues introduced to replace the correct residues are in red. **(C)** The PIAL2 protein harboring the SIM mutations interacts with MOM1 but not with SUMO2 as shown by yeast two-hybrid assays. MOM1 and PIAL2 were fused with GAL4-AD. The PIAL2 sequence harboring the SIM mutations was fused with GAL4-BD. “Vec” represents the empty *GAL-BD* or *GAL4-AD* vectors.
(PDF)

S5 Fig The expression levels of the wild-type and the mutated *MOM1-Flag* transgenes in the *mom1* mutant background. **(A)** The mutations (V1994A/V1995A/L1997A/S1998A) in the CMM3 domain encoded by the mutated *MOM1-Flag* transgene. The mutated residues were shown in blue. The Ala residues introduced to replace the correct residues were shown in red. **(B)** The expression of the wild-type and mutated MOM1-Flag transgenes was determined by western blotting. The loading of proteins was indicated by Ponceau S staining. The transgenic lines were used for complementation testing.
(PDF)

S1 Table Primers used in this study. (XLSX)
