# Supplementary material for: Exploring potential roles for the interaction of MOM1 with SUMO and the SUMO E3 ligase-like protein PIAL2 in transcriptional silencing
Source: PLoS One. 2018 Aug 9;13(8):e0202137. doi: 10.1371/journal.pone.0202137 (PMC6084981; doi:10.1371/journal.pone.0202137)
Supplement: S1 Fig — (A) Schematic representation of the Arabidopsis PIAL2 protein. (B) Alignment of PIAL2 and its homologs in Brassica rapa, Populus trichocarpa and Vitis vinifera. The IND domain of PIAL2 is from 143 to 201 amino acids. The SIM domain is from 425 to 428 amino acids. (PDF) [file pone.0202137.s001.pdf]

Supplemental Figure 1

A

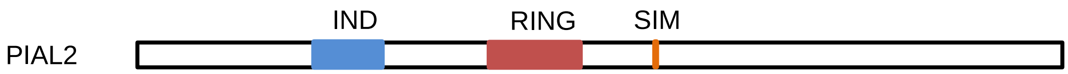

B

```
Arabidopsis         ---------MSTAAAARPVAGTGLREKTAASLVNSFRLASVTQRLRYHIQDGAKVDPKEFQICCISFAKGIDFAIANNDI  71
Brassica rapa       --------MSSASTAVRPVAGTGFPGKAAAALVNSFRLASVTQRLAFHIQTGAKSDVKEFQSCCISLAKGIDFAIANNEI  72
populus euphratica  MAGMMAGTMMPTPQAAEGVTIGAAGQQMSASLANSFRVHAVAERLSLHIQPGFDSNPTEFFGLCLSLARGIDFAVANNET  80
Vitis vinifera      MAGM---------------SYTDDGGRG-RIRPASRLVATVADLLAMHIQNGHPLDSAEVADLFLSLARGIDHAVANNEI  64

Arabidopsis         PKKVEEFPWLLKQLCRHGTDVYTKTALMVLMISVKHACHLGWFSDSESQELIALADEIRTCFGSSGSTSPGIKSPGSTFS  151
Brassica rapa       PKKVEDLPSLLKQVCRHRDDVYTKTAVLVLMISIKHACKLGWFSDSEAQELIALADQMKNGFGNPENTIPVIQSPGGTLS  152
populus euphratica  LLKAQELPFLLKQVCQRKNDVFLQAAIMVLMASVKNACMVGWFQEKETQELVTLATEIGKVFCTPGDINAGTTDSLSIIS  160
Vitis vinifera      PSRARDLPFLLKQVLRRMNDSSLQAVAMVLMISVKNACKIGWFLDHDATDLLTLAKEIGKIFSTMEDINAEPHYPLPSVS  144

Arabidopsis         QIMERFYPFVKLGHVLVSFEVKAGYTMLAHDFYISKNMPHSLQEKIRLFVAQTDNIDTSACISNPPEVSFLLNGKGVEKR  231
Brassica rapa       QIMERFYPLVKLGHVLVSLEVKSGYTMLAHDFHISKNMPHSPQEKIRLFVVQTENIDTSACIINPPEVSFILNGKVVEKR  232
populus euphratica  TIMSRFYPLMKMGQIIVSLEVKPGFGAHVIDFHISKTTRNLTEDKIWLFVAQTDNTETSTCIVTPQEVNFLLNGKGVERR  240
Vitis vinifera      KIMSRYYPRLRMGHVLASLEVKPGYGAFVIDFHITRSMVSPAQKHICLFVAQTDNMDTSSCIVTPPQVNFLLNGKGVWGR  224

Arabidopsis         VNIAMDTGPQLPTNVTAQLKYGTNLLQVMGNFKGNYIIIIAFTGLVVPPEKPVLKDYLQSGVIEASPDSDIIEGPSRVSL  311
Brassica rapa       VNISMDSGPQLPTNVTAILKYGTNLLQVMGNSNGHYIIVIAFTGLAQLPEKPVLKEYVQSGVVEASPDSDIIEGPSRVSL  312
populus euphratica  TNVIMDTGPQMPTNVTGMLKYGTNLLQAVGQFKGHYVIAVAFMSVEPKPETPVLQDYVHPSAAELDPDSDIIEGPSRISL  320
Vitis vinifera      INVSMDNGPQLPTNVIAMLRYGINLLQVVGQFNGNYVIIIAFMSVISTSGIPELQEYIQPVAVTSDSDLEIIEGQARISL  304

Arabidopsis         SCPISRKRIKLPVKGQLCKHLQCFDFSNYVHINMRNPTWRCPHCNQPVCYPDIRLDQNMAKILKDVEHNAADVIIDAGGT  391
Brassica rapa       RCPISRSRIKLPVKGQLCKHLQCFDFWNYVNINMRNPSWRCPHCNQPVSYPEIRLDQNMVEILKDAGRNAADVVIHAGGT  392
populus euphratica  NCPISYTRIRTPVKGHSCRHLQCFDFSNFVDINTRRPSWRCPHCNQHVCYTDIRIDQNMVKVLREVGDHCSVVIISADGS  400
Vitis vinifera      NCPISFRRINIPVKGHLCKHHQCFDYGNFIEINSRRPSWRCPHCNQSVCNPDIRIDQNMVKILREVGENVVDVIISPDGS  384

Arabidopsis         WKVTKNTGETPEPVR-EIIHDL------EDPMSLLNSGPVVFDLTGDDD--AELEVFGDN-KVEDRKPCMSDAQGQSNNN  461
Brassica rapa       WKVAMENNGNEEPVRDAIIHDL------EDPNSLLNAGPVVLDLTGDDEDDADIELFGSNTKAVDQKPHLSDAQGQSNNN  466
populus euphratica  LKAISESDNKVDQTQERTLHCDKSMPEQVESMTSMRALPVVMDLTVD-DDDING---KDNIDAEDRKPFLATLQNHPVDT  476
Vitis vinifera      WKPVVESIDHAEQLYDATQSNWQENTKQCESVRLSSIAADAVDLTMGEDNDDDS---PSNFRTEDMKPLWDDLQGFSSAE  461

Arabidopsis         NTNKHPSN----------------DD-YSSIFDISDV-----------------I----------ALDPEILSAL-----  492
Brassica rapa       NTSKDASV----------------DD-YCSMFNFSDV-----------------I----------SLDEVMLDH------  496
populus euphratica  NPIPTMPSQLVNTNAPSRNFSTLADEFWSSPYWSSSAS-----------DAQMVNGFSEPSTTTFMTSPVITDSVSPALN  545
Vitis vinifera      KIVSPGVNSTV--EADQIISAHREDNLWTGVLLTPSSVSDGLAPPTTSSNAHSNVGF-PRSTFSFMSSPVLTDAVSPSPY  538

Arabidopsis         ---G------NT---------------APQPHQASNTGTGQQYSNLSQIPMSIDPMPVPVP----FSQTPSPRDRPATTS  544
Brassica rapa       ----------------------------------LNTGTGQDYLNLSQVPMPRDPAQVPAP----FSQAPSPRERSATTS  538
populus euphratica  CDVGGY---GNTTTSVMHNQLSASSYLQSLQQKFVNSVANGEYGTLPPI-YHVNRSPIAVQALPARPQTPAPQQRSRTPN  621
Vitis vinifera      RETLDVHRETQVPIPLLQNQHFDPSNLQLQQSRLGSLIASNEYGRLASIPRHLTRNPIAVQALPAQDQLPRLAQHTRLMP  618

Arabidopsis         TVF-----------TIPNPSPQYSQVHASPVTPTGTY------LGRTTSPRWNQ---------TYQSQAPP---------  589
Brassica rapa       TVF-----------PS-------SRVHASPVTPAGTY------LSRTSSQMSS---------------------------  567
populus euphratica  P-AISSGASLSSHGTLPEAANGLSSVSG--NMDRQQQFARSLNMNSSSSQNWNLQDHPFMHGQSAQQQAVTLPSSSQLAG  698
Vitis vinifera      TGATSTGSQ--TTSFMAPSVEGFDAVNG--VTERDLQFSRSLMSSFPV------------SGQSVQR-VGGLP-------  674

Arabidopsis         --------------MTTPYTSRKVSVPVTSQSP----------------ANVSSFVQS-------------------QHV  620
Brassica rapa       --------------LTTSSQSRVHPVQVTSQSL----------------GNGSSLAQS-------------------PRI  598
populus euphratica  AHRASSPNLLYQQPLRVP-QSRSHSPNVVRSSLPLAPAL--TQQGAAQVGNSAGATNSQQSRLMVAAQLVAQRARQPPSV  775
Vitis vinifera      -------NPRTTQAMNEP-RNNVH------PSIHVQSMQRQQRSGGSQ---VTGSVPNRQSPHAAAAQQTVQVSRSPPSV  737

Arabidopsis         PRVLS-QPNNYGVRGLTS---SH---ASTSRQHPSGPTVQSVSRLSDLVDVDLTVPDTSNWRP--RMRGSLVPGSHSTAL  691
Brassica rapa       PRVLGPQPNSYFARSLNS---NH---LTTQTQRPSSPPVLSVSRTSDLMDVDSATPDTSNWRP--RMRGSITPGSYSPAL  670
populus euphratica  PVQIQ--TSGAGASYLTSA--DGIRAPATEQRGNAGGALPAVSGTE----GLVDLASEQNWRPTGRMRGSLSGRAYSAAL  847
Vitis vinifera      PVQLR--PARTGTAFSVGMVAEQLRTA-GEQRRNILGTAWSTPRPD----ASAALPTDENWRPSGRMRGSLTGEAYSAAL  810

Arabidopsis         DHMIIRPSQQSQTSTR---LNSSQPVQTPSVQTSQAQSPFTTAAYRTETVLGNRNHPVPAPPGIVRPT-----------G  757
Brassica rapa       DHMIIRPTQPSQT--R---LQVSQPGQTPPVQTSQALPPFSTA-----------------PPTFTRPS-----------G  717
populus euphratica  KEFMVQPTQQTQTPRPPPNLPPSQSSMPPHLQFL-----------------FARNAQVPQ--AQSSPV-SGSAISNGSSS  907
Vitis vinifera      NQFMLQPTQPTQAPLPPTSLPSNLPGGLPAFRLF-----------------PSSSNEHNAAPSQTEPQPRLRPQGGGSSG  873

Arabidopsis         PTS-------  760
Brassica rapa       PTAPWGT---  724
populus euphratica  TLP-------  910
Vitis vinifera      TLPEWTPRMP  883
```

S1 Fig. Analysis of the Arabidopsis PIAL2 protein. (A) Schematic representation of the Arabidopsis PIAL2 protein. **(B)** Alignment of PIAL2 and its homologs in *Brassica rapa, Populus trichocarpa and Vitis vinifera*. The IND domain of PIAL2 is from 143 to 201 amino acids. The SIM domain is from 425 to 428 amino acids.
