# Supplementary material for: Exploring potential roles for the interaction of MOM1 with SUMO and the SUMO E3 ligase-like protein PIAL2 in transcriptional silencing
Source: PLoS One. 2018 Aug 9;13(8):e0202137. doi: 10.1371/journal.pone.0202137 (PMC6084981; doi:10.1371/journal.pone.0202137)
Supplement: S2 Fig — Both the wild-type (PIAL2-WT) and mutated (PIAL2-IND-M) transgenes were driven by the native PIAL2 promoter and tagged by Myc epitope in their C-terminals. The expression of the transgenes was detected by western blotting. Rubisco stained by Ponceau S was shown as a loading control. (PDF) [file pone.0202137.s002.pdf]

Supplemental Figure 2

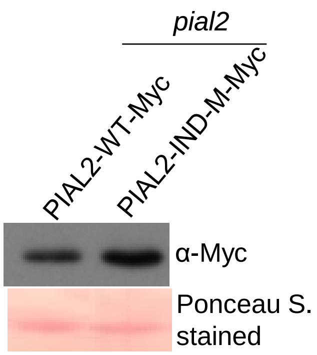

S2 Fig. Determination of the expression of the wild-type and mutated *PIAL2* transgenes. Both the wild-type (*PIAL2-WT*) and mutated (*PIAL2-IND-M*) transgenes were driven by the native *PIAL2* promoter and tagged by Myc epitope in their C-terminals. The expression of the transgenes was detected by western blotting. Rubisco stained by Ponceau S was shown as a loading control.
