# Supplementary material for: Exploring potential roles for the interaction of MOM1 with SUMO and the SUMO E3 ligase-like protein PIAL2 in transcriptional silencing
Source: PLoS One. 2018 Aug 9;13(8):e0202137. doi: 10.1371/journal.pone.0202137 (PMC6084981; doi:10.1371/journal.pone.0202137)
Supplement: S3 Fig — The transgenes were introduced into the mom1/pial2 double mutant and their expression was determined by western blotting. Rubisco stained by Ponceau S was shown as a loading control. (PDF) [file pone.0202137.s003.pdf]

Supplemental Figure 3

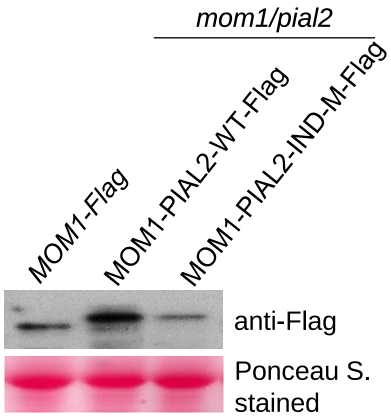

S3 Fig. Determination of the expression of the *MOM1-Flag* transgene, the wild-type *MOM1-PIAL2* fusion transgene, and the *MOM1-PIAL2* fusion transgene harboring the mutations in the *IND* domain of *PIAL2*. The transgenes were introduced into the *mom1/pial2* double mutant and their expression was determined by western blotting. Rubisco stained by Ponceau S was shown as a loading control.
