# Supplementary material for: Exploring potential roles for the interaction of MOM1 with SUMO and the SUMO E3 ligase-like protein PIAL2 in transcriptional silencing
Source: PLoS One. 2018 Aug 9;13(8):e0202137. doi: 10.1371/journal.pone.0202137 (PMC6084981; doi:10.1371/journal.pone.0202137)
Supplement: S4 Fig — (A) PIAL2 interacts with SUMO2 as determined by yeast two-hybrid assays. PIAL1 and PIAL2 were fused with GAL4-AD. SUMO2 were fused with GAL4-BD. “Vec” represents the empty GAL-BD or GAL4-AD vectors. (B) Schematic representation of mutations in the SIM domain of PIAL2. The mutated Val, Phe, Asp, and Leu residues of PIAL2 are in blue. The Ala residues introduced to replace the correct residues are in red. (C) The PIAL2 protein harboring the SIM mutations interacts with MOM1 but not with SUMO2 as shown by yeast two-hybrid assays. MOM1 and PIAL2 were fused with GAL4-AD. The PIAL2 sequence harboring the SIM mutations was fused with GAL4-BD. “Vec” represents the empty GAL-BD or GAL4-AD vectors. (PDF) [file pone.0202137.s004.pdf]

Supplemental Figure 4

A

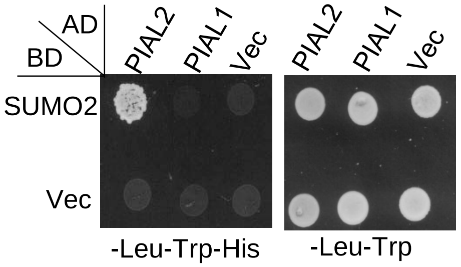

C

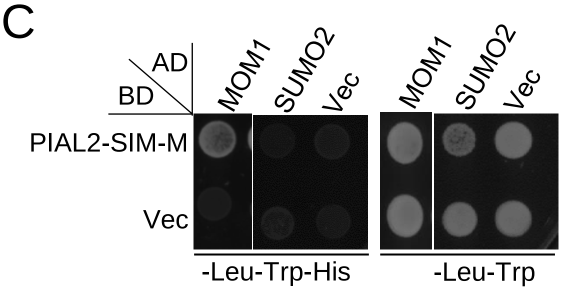

B

SIM-M: AAAA

PIAL2: VREIIHDLEDPMSLLN SGPVVFDLTGDDDAELE 437 aa

S4 Fig. The SIM domain of PIAL2 is responsible for PIAL2 interaction with SUMO2 but not with MOM1. (A) PIAL2 interacts with SUMO2 as determined by yeast two-hybrid assays. PIAL1 and PIAL2 were fused with GAL4-AD. SUMO2 were fused with GAL4-BD. “Vec” represents the empty *GAL-BD* or *GAL4-AD* vectors. **(B)** Schematic representation of mutations in the SIM domain of PIAL2. The mutated Val, Phe, Asp, and Leu residues of PIAL2 are in blue. The Ala residues introduced to replace the correct residues are in red. **(C)** The PIAL2 protein harboring the SIM mutations interacts with MOM1 but not with SUMO2 as shown by yeast two-hybrid assays. MOM1 and PIAL2 were fused with GAL4-AD. The PIAL2 sequence harboring the SIM mutations was fused with GAL4-BD. “Vec” represents the empty *GAL-BD* or *GAL4-AD* vectors.
