# Supplementary material for: Exploring potential roles for the interaction of MOM1 with SUMO and the SUMO E3 ligase-like protein PIAL2 in transcriptional silencing
Source: PLoS One. 2018 Aug 9;13(8):e0202137. doi: 10.1371/journal.pone.0202137 (PMC6084981; doi:10.1371/journal.pone.0202137)
Supplement: S5 Fig — (A) The mutations (V1994A/V1995A/L1997A/S1998A) in the CMM3 domain encoded by the mutated MOM1-Flag transgene. The mutated residues were shown in blue. The Ala residues introduced to replace the correct residues were shown in red. (B) The expression of the wild-type and mutated MOM1-Flag transgenes was determined by western blotting. The loading of proteins was indicated by Ponceau S staining. The transgenic lines were used for complementation testing. (PDF) [file pone.0202137.s005.pdf]

Supplemental Figure 5

A

```
 MOM1-CMM3-M: SLSSGLQSNNEAACAADDE
MOM1-CMM3-WT: SLSSGLQSNNEVVCLSDDE
```

B

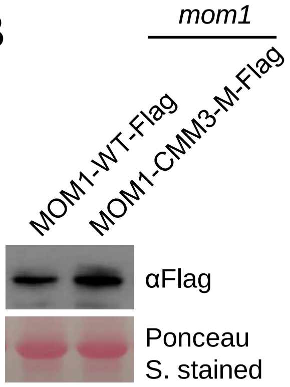

S5 Fig The expression levels of the wild-type and the mutated *MOM1-Flag* transgenes in the *mom1* mutant background. (A) The mutations (V1994A/V1995A/L1997A/S1998A) in the CMM3 domain encoded by the mutated *MOM1-Flag* transgene. The mutated residues were shown in blue. The Ala residues introduced to replace the correct residues were shown in red. **(B)** The expression of the wild-type and mutated MOM1-Flag transgenes was determined by western blotting. The loading of proteins was indicated by Ponceau S staining. The transgenic lines were used for complementation testing.
